# Cognitive Diagnosis Modeling Incorporating Item-Level Missing Data Mechanism

**DOI:** 10.3389/fpsyg.2020.564707

**Published:** 2020-11-30

**Authors:** Na Shan, Xiaofei Wang

**Affiliations:** ^1^School of Psychology, Northeast Normal University, Changchun, China; ^2^Key Laboratory of Applied Statistics of the Ministry of Education, Northeast Normal University, Changchun, China; ^3^School of Mathematics and Statistics, Northeast Normal University, Changchun, China

**Keywords:** cognitive diagnosis, item-level, missing data, missing data mechanism, cognitive diagnosis model

## Abstract

The aim of cognitive diagnosis is to classify respondents' mastery status of latent attributes from their responses on multiple items. Since respondents may answer some but not all items, item-level missing data often occur. Even if the primary interest is to provide diagnostic classification of respondents, misspecification of missing data mechanism may lead to biased conclusions. This paper proposes a joint cognitive diagnosis modeling of item responses and item-level missing data mechanism. A Bayesian Markov chain Monte Carlo (MCMC) method is developed for model parameter estimation. Our simulation studies examine the parameter recovery under different missing data mechanisms. The parameters could be recovered well with correct use of missing data mechanism for model fit, and missing that is not at random is less sensitive to incorrect use. The Program for International Student Assessment (PISA) 2015 computer-based mathematics data are applied to demonstrate the practical value of the proposed method.

## 1. Introduction

Cognitive diagnosis has recently received increasing concern in psychological and educational assessment, which can provide fine-grained classifications and diagnostic feedback for respondents from their performance on test items (Leighton and Gierl, 2007; Rupp et al., 2010). It is a useful tool to identify students' mastery status of different latent skills based on their responses to test items and to evaluate patients' presence of mental disorders based on their responses to diagnostic questions. More specifically, cognitive diagnosis has been used to study fraction subtraction (de la Torre and Douglas, 2004), language proficiency (von Davier, 2008; Chiu and Köhn, 2016), psychological disorders (Templin and Henson, 2006; Peng et al., 2019), and so forth.

Various cognitive diagnosis models (CDMs), also called diagnosis classification models, have been developed, such as the deterministic inputs, noisy “and” gate (DINA) model (Macready and Dayton, 1977; Junker and Sijtsma, 2001) and the deterministic inputs, noisy “or” gate (DINO) model (Templin and Henson, 2006). Most CDMs are parametric and model the probability of item response as a function of latent attributes. The simplicity and interpretability make the parametric CDMs popular in practice. More general CDMs, such as the log-linear cognitive diagnosis model (LCDM; Henson et al., 2009) and the generalized DINA model (de la Torre, 2011), assume a more flexible relationship between the item responses and latent attributes. Moreover, higher-order latent trait models for cognitive diagnosis (de la Torre and Douglas, 2004) have been introduced to link the correlated latent traits by a general high-order ability.

Multiple items are often used for cognitive diagnosis. When respondents choose to answer some but not all items, item-level missing data occur (Chen et al., 2020). Respondents may refuse to answer items that they deem too difficult, quit the test early because it is too long, or just skip items because of carelessness. Missing data lead to loss of information and may result in biased conclusions (Glas and Pimentel, 2008; Köhler et al., 2015; Kuha et al., 2018). Many studies have employed a complete case analysis that only use subjects without missing data (e.g., Xu and Zhang, 2016; Chen et al., 2017; Zhan et al., 2019a). In this case, the subjects with missing data cannot receive any diagnostic feedback and, more importantly, may produce biased results when subjects with complete data are systematically different from those with missing data (Pan and Zhan, 2020).

In the literature, three missing data mechanisms should be distinguished: missing completely at random (MCAR), missing at random (MAR), and missing not at random (MNAR) (Rubin, 1976; Little and Rubin, 2020). The MCAR holds if the probability of missingness is independent of both the observed and unobserved responses, whereas the MAR holds if the probability of missingness is independent of the unobserved responses given the observed responses. If either of these two conditions cannot be satisfied, i.e., the probability of missingness depends on the unobserved responses, the MNAR occurs. If the missing data mechanism is MCAR or MAR, unbiased estimation can be obtained from the observed data; if the missing data mechanism is MNAR, a model for the missing data mechanism should be included to obtain valid estimations of the primary parameters. Limited approaches have been proposed in CDMs incorporating missing data mechanisms. Ömür Sünbül (2018) considered MCAR and MAR in the DINA model. Recently, Ma et al. (2020) have used the sequential process model to accommodate omitted items due to MNAR, where the first internal task, i.e., making the decision to either skip the item or respond to it, is assumed to be affected by a latent categorical variable representing the response tendency. As stated by Heller et al. (2015), CDMs have connections to knowledge space theory (KST), which has been developed by Doignon and Falmagne (1985) (see also Doignon and Falmagne, 1999; Falmagne and Doignon, 2011). de Chiusole et al. (2015) and Anselmi et al. (2016) have developed models for the analysis of MCAR, MAR, and MNAR data in the framework of KST. In their work, the MCAR holds if the missing response pattern is independent of the individual's knowledge state (i.e., the collection of all items that an individual is capable of solving in a certain disciplinary domain) and of the observed responses; the MAR holds if the missing response pattern is conditionally independent of the knowledge state given the observed responses; and the MNAR holds if the missing response pattern depends on the knowledge state. In CDMs, the attribute profile (i.e., the collection of all attributes that an individual masters in a certain disciplinary domain) is similar to the knowledge state in KST. In our study, an additional latent categorical variable is introduced to indicate missingness propensity. This latent categorical variable affects the probability of missingness for each item and, in the meantime, may affect the latent attributes through the influence of the general high-order ability. The missing data mechanism can then be incorporated into cognitive diagnosis. Similar ideas can be seen in Holman and Glas (2005), Rose et al. (2015), and Kuha et al. (2018).

In this paper, we propose a joint cognitive diagnosis modeling including a higher-order latent trait model for item responses and a missingness propensity model for item-level missing data mechanism. We take the DINA model as an example for illustration because of its popular use, and the latent traits are linked by a general high-order ability. For a flexible specification of the missingness propensity model, the latent missingness propensity is represented by a categorical variable. The MNAR holds if the distribution of the general high-order ability depends on the latent classes, whereas the MCAR holds if the distribution of the general high-order ability is independent of the latent classes.

The rest of this paper is organized as follows. Section 2 presents the proposed joint model for item responses and item-level missing data mechanism. The Bayesian approach is then developed for model parameter estimation using JAGS. In section 3, simulation studies are conducted to compare the performance of the parameter recovery under different missing data mechanisms. Real data analysis using the PISA 2015 computer-based mathematics data is given in section 4. Some concluding remarks are given in section 5.

## 2. Joint Modeling Incorporating Item-Level Missing Data Mechanism

We consider *N* subjects taking a test of *I* items, and there are *K* latent attributes to be evaluated. Let *Y*_*ni*_ be the response for subject *n*(*n* = 1, ⋯ , *N*) to item *i*(*i* = 1, ⋯ , *I*). Let *R*_*ni*_ be a missingness indicator corresponding to *Y*_*ni*_, where *R*_*ni*_ = 1 if *Y*_*ni*_ is observed and *R*_*ni*_ = 0 if *Y*_*ni*_ is missing.

### 2.1. The Missingness Propensity Model

Let ξ_*n*_ denote the latent missingness propensity for subject *n*. ξ_*n*_ is unobserved and has *C* categories, which we refer to as latent missingness classes. The missingess probability of (*R*_*n*1_, ⋯ , *R*_*nI*_) is given by

(1) $$p\left(R_{n1},\cdots \;,R_{nI}\right)=\overset{C}{\underset{c=1}{\sum }}\left\{\overset{I}{\underset{i=1}{\prod }}p\left(R_{ni}|\xi_{n}=c\right)\right\}p\left(\xi_{n}=c\right),$$

where *p*(*R*_*n*1_, ⋯ , *R*_*nI*_) is the joint probability of (*R*_*n*1_, ⋯ , *R*_*nI*_), *p*(*R*_*ni*_ | ξ_*n*_ = *c*) is the conditional probability of *R*_*ni*_ given ξ_*n*_ = *c* and *p*(ξ_*n*_ = *c*) is the probability of ξ_*n*_ = *c*.

The latent missingness propensity ξ_*n*_ is specified as the categorical distribution

(2) $$\xi_{n}\sim \text{Categorical}\left(\pi \right),$$

where ***π*** = (π_1_, ⋯ , π_*C*_) and $\Sigma_{c=1}^{C}\pi_{c}=1$. The conditional probability of *R*_*ni*_ is specified as the logistic function

(3) $$\text{logit}\left\{p\left(R_{ni}=1|\xi_{n}=c\right)\right\}=\tau_{0i}+\overset{C}{\underset{c=2}{\sum }}\tau_{ci}\xi_{\left(c\right)},$$

where τ_0*i*_ and τ_*ci*_(*c* = 2, ⋯ , *C*) are the intercept and slope parameters, and ξ_(*c*)_(*c* = 2, ⋯ , *C*) are dummy variables for the missingness classes. A positive slope parameter τ_*ci*_ is assumed, which means that the missingness probability reduces in the latter missingness class compared to the first class. Denote ***τ***_0_ = (τ_01_, ⋯ , τ_0*I*_) and ***τ***_*c*_ = (τ_*c*1_, ⋯ , τ_*cI*_) for *c* = 2, ⋯ , *C*. The idea of introducing latent variables to model the non-response mechanism have been proposed previously by, for example, Lin et al. (2004) and Hafez et al. (2015). The missingness propensity model is identifiable when *C* ≤ 2^*I*^/(1 + *I*).

### 2.2. The High-Order DINA Model

The DINA model describes the probability of item response as a function of latent attributes as follows:

(4) $$p\left(Y_{ni}=1\right)=g_{i}+\left(1-s_{i}-g_{i}\right)\overset{K}{\underset{k=1}{\prod }}\alpha_{nk}^{q_{ik}},$$

where *p*(*Y*_*ni*_ = 1) is the probability of a correct response for subject *n* to item *i*; *s*_*i*_ and *g*_*i*_ are the slipping and guessing probability for item *i* respectively, and 1 − *s*_*i*_ − *g*_*i*_ is the item discrimination index for item *i* (IDI_*i*_; de la Torre, 2008), α_*nk*_ is the *k*th (*k* = 1, ⋯ , *K*) latent attribute for subject *n*, with α_*nk*_ = 1 if subject *n* masters attribute *k* and α_*nk*_ = 0 otherwise. The *Q* matrix is a *I* × *K* matrix with binary entries *q*_*ik*_ (Tatsuoka, 1983). For each *i* and *k*, *q*_*ik*_ = 1 indicates that attribute *k* is required to answer item *i* correctly and *q*_*ik*_ = 0 otherwise.

Equation (4) can be reparameterized, and it is called the reparameterized DINA model (DeCarlo, 2011) as

(5) $$\text{logit}\left(p\left(Y_{ni}=1\right)\right)=\beta_{i}+\delta_{i}\overset{K}{\underset{k=1}{\prod }}\alpha_{nk}^{q_{ik}},$$

where β_*i*_ = logit(*g*_*i*_) and δ_*i*_ = logit(1 − *s*_*i*_) − logit(*g*_*i*_) are called item intercept and interaction parameter, respectively.

As stated in the literature (de la Torre and Douglas, 2004; Zhan et al., 2018a), attributes in a test are often correlated, and a higher-order structure for the attributes can be formulated by

(6) $$\text{logit}\left(p\left(\alpha_{nk}=1\right)\right)=\gamma_{k}\theta_{n}-\lambda_{k},$$

where *p*(α_*nk*_ = 1) is the probability of subject *n*'s mastery of attribute *k*; θ_*n*_ is a general (higher-order) ability for subject *n*; and γ_*k*_ and λ_*k*_ are the slope and intercept parameter for attribute *k*. Denote ***γ*** = (γ_1_, ⋯ , γ_*K*_) and ***λ*** = (λ_1_, ⋯ , λ_*K*_). Following Zhan et al. (2018a), a positive slope parameter is assumed, which means the probability of mastery of attribute *k* increases as the general ability θ_*n*_ grows. Including a higher-order structure for cognitive diagnosis can not only reduce the number of model parameters for correlated latent attributes but also obtain an assessment for subjects' overall ability.

The distribution of the general ability θ_*n*_ may be affected by the latent missingness classes of ξ_*n*_, and we suppose that

(7) $$\theta_{n}|\xi_{n}=c\sim N\left(\mu_{c},\sigma_{c}^{2}\right),$$

for *n* = 1, ⋯ , *N* and *c* = 1, ⋯ , *C*. If μ_*c*_ or $\sigma_{c}^{2}$ (*c* = 1, ⋯ , *C*) may vary between different classes, the missing data mechanism is MNAR, and we set μ_1_ = 0 and $\sigma_{1}^{2}=1$ for model identification. If μ_*c*_ and $\sigma_{c}^{2}$ remain unchanged for different *c*, the probability of missingness does not depend on the responses and MCAR holds, and this is where we set θ_*n*_ ~ *N*(0, 1) for identification.

### 2.3. Bayesian Parameter Estimation

The parameters of the proposed model can be estimated using the Bayesian MCMC approach. JAGS (version 4.2.0; Plummer, 2015) and the R2jags package (Su and Yajima, 2020) in R (R Core Team, 2019) were used for estimation, and the JAGS code can be found in the Supplementary Material. The priors of the model parameters are given below. For the majority of the parameters, the conjugate priors are used. The priors and the hyper priors for the item parameters are assigned the same as those given in Zhan et al. (2018a), please find them for details. Moreover, the noninformative prior is used for Dirichlet distribution. The (truncated) normal distribution and the inverted gamma distribution priors are chosen to obtain dispersed values for each corresponding parameter.

$$\left(\pi_{1},\cdots \;,\pi_{C}\right)\sim \text{Dirichlet}\left(1,\cdots \;,1\right),$$

$$\mu_{c}\sim N\left(0,4\right),\;\sigma_{c}^{2}\sim \text{InvGamma}\left(1,1\right),\;\left(c=2,\cdots \;,C\right),$$

$$\gamma_{k}\sim N\left(0,4\right)I\left(\gamma_{k}>0\right),\;\lambda_{k}\sim N\left(0,4\right),\;\left(k=1,\cdots \;,K\right),$$

$$\begin{array}{r} \tau_{0i}\sim N\left(0,4\right),\;\tau_{ci}\sim N\left(0,4\right)I\left(\tau_{ci}>0\right),\\ \;\left(i=1,\cdots \;,I;c=2,\cdots \;,C\right), \end{array}$$

$$\begin{array}{l} \left(\begin{array}{c} \beta_{i}\\ \delta_{i} \end{array}\right)\sim N\left(\left(\begin{array}{c} \mu_{\beta }\\ \mu_{\delta } \end{array}\right),\Sigma_{\text{item}}\right),\;\left(i=1,\cdots \;,I\right). \end{array}$$

The hyper priors are specified:

$$\begin{array}{r} \mu_{\beta }\sim N\left(-2.197,2\right),\mu_{\delta }\sim N\left(4.394,2\right)I\left(\mu_{\delta }>0\right),\Sigma_{\text{item}}\\ \sim \text{InvWishart}\left(I,2\right), \end{array}$$

where ***I*** is a 2 × 2 identity matrix. In this case, the mean guessing and slipping probabilities are approximately equal to 0.1.

In this paper, the number of latent missingess classes *C* is taken as fixed. In fact, *C* can be selected by some information criterion, for example, deviance information criterion (DIC; Spiegelhalter et al., 2002), which can result in a statistically optimal number. In practice, it is efficient to determine *C* beforehand, using latent class analysis (Linzer and Lewis, 2011) just for the missingness indicators. In the following simulation studies, the number of latent missingess classes *C* is fixed to 2 for simplicity. For the values of *C* > 2, the results are similar and we report some results for *C* = 3 in the Supplementary Material.

## 3. Simulation Study

Two simulation studies were conducted to evaluate the empirical performance of the proposed method. Simulation 1 aimed to examine the parameter recovery using the Bayesian MCMC algorithm when the simulated data were generated under MNAR. Under different conditions, models with MCAR and MNAR were fitted to the simulated data, respectively, where the distribution of the general higher-order ability was unrelated or related to the latent missingness classes in MCAR or MNAR. In Simulation 2, our purpose was to study the sensitivity of incorrect use of MNAR for model fit. Parameter recovery related to diagnostic classification are reported here. The other parameters about the missing data mechanisms can be recovered well but not reported here since they are not our primary interest.

### 3.1. Simulation Study 1

**Figure 1:**
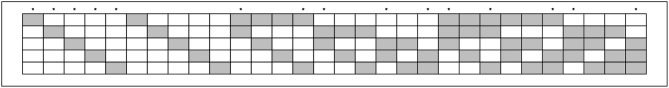
*K*-by-*I Q* matrix for simulation study 1. Blank means “0,” gray means “1”; “*” denotes items used in *I* = 15 conditions; *K* = the number of attributes; *I* = test length.

In Simulation 1, three factors were manipulated, including (*a*) sample sizes (*N*) at two levels of 500 and 1,000; (*b*) test length (*I*) at two levels of 15 and 30; and (*c*) the probability of missingness for each item, high missingness (HM) and low missingness (LM). Five attributes (*K* = 5) were measured and the simulated *Q* matrices for two test length *I* = 15 and *I* = 30 were given in Figure 1, which were used in Zhan et al. (2018b). Most of the model parameters were assigned by referring to the real data analysis presented in Zhan et al. (2018a). Specifically, ***π*** = (0.3, 0.7), representing unequal probabilities for each latent class; τ_2*i*_ = 1.2 for all items, ***τ***_0_ = (0.0, 0.5, 0.0, 0.5, 0.0, 0.5, 0.0, 0.5, 0.0, 0.5, 0.0, 0.5, 0.0, 0.5, 0.0) for high missingness with *I* = 15, ***τ***_0_ = (0.0, 0.5, 0.0, 0.5, 0.0, 0.5, 0.0, 0.5, 0.0, 0.5, 0.0, 0.5, 0.0, 0.5, 0.0, 0.5, 0.0, 0.5, 0.0, 0.5, 0.0, 0.5, 0.0, 0.5, 0.0, 0.5, 0.0, 0.5, 0.0, 0.5) for high missingness with *I* = 30, ***τ***_0_ = (1.0, 1.5, 1.0, 1.5, 1.0, 1.5, 1.0, 1.5, 1.0, 1.5, 1.0, 1.5, 1.0, 1.5, 1.0) for low missingness with *I* = 15 and ***τ***_0_ = (1.0, 1.5, 1.0, 1.5, 1.0, 1.5, 1.0, 1.5, 1.0, 1.5, 1.0, 1.5, 1.0, 1.5, 1.0, 1.5, 1.0, 1.5, 1.0, 1.5, 1.0, 1.5, 1.0, 1.5, 1.0, 1.5, 1.0, 1.5, 1.0, 1.5) for low miss -ingness with *I* = 30, corresponding to the missingness probability for each item between 0.22 and 0.31 in the case of high missingness and between 0.1 and 0.22 in the case of low missingness; μ_2_ = 1.0 and $\sigma_{2}^{2}=0.5$; γ_*k*_ = 1.5 for all attributes and ***λ*** = (−1.0, −0.5, 0.0, 0.5, 1.0), indicating moderate correlations between attributes; μ_item_ = (μ_β_, μ_δ_) = (−2.197, 4.394) and

$$\begin{array}{l} \Sigma_{\text{item}}=\left(\begin{array}{cc} \Sigma_{11} & \Sigma_{12}\\ \Sigma_{12} & \Sigma_{22} \end{array}\right)=\left(\begin{array}{cc} 1.0 & -0.8\\ -0.8 & 1.0 \end{array}\right), \end{array}$$

which was the mean vector and the covariance matrix for a bivariate normal distribution generating β_*i*_ and δ_*i*_. Other assignments for the model parameters, for example, equal probabilities for each latent class as those used in Ma et al. (2020), also make sense, and the results are similar to the above parameter settings.

In each of the eight conditions, models with MCAR and MNAR were fitted to the simulated data, respectively. Thirty replications were implemented for each fitted model. Pilot runs showed that the algorithm converged using 20, 000 iterations with a burn-in phase of 10, 000 iterations. The convergence of the chains was monitored by multivariate potential scale reduction factor, which were < 1.1 (Gelman and Rubin, 1992). The bias and the root mean square error (RMSE) of the Bayesian estimates were computed to assess the parameter recovery. For evaluating the classification of each attribute and attribute profiles, the attribute correct classification rate (ACCR) and the pattern correct classification rate (PCCR) were computed. More formally, if the *k*th (*k* = 1, ⋯ , *K*) latent attribute for subject *n* (*n* = 1, ⋯ , *N*), denoted as α_*k,n*_, is estimated by ${\hat{\alpha }}_{k,n}$, ACCR for the *k*th latent attribute and PCCR can be expressed as $\text{ACCR}=\overset{N}{\underset{n=1}{\sum }}I\left({\hat{\alpha }}_{k,n}=\alpha_{k,n}\right)/N$ and $\text{PCCR}=\overset{N}{\underset{n=1}{\sum }}\left[\prod_{k=1}^{K}I\left({\hat{\alpha }}_{k,n}=\alpha_{k,n}\right)\right]/N$, respectively. Here $I\left({\hat{\alpha }}_{k,n}=\alpha_{k,n}\right)$ is an indicator function such that $I\left({\hat{\alpha }}_{k,n}=\alpha_{k,n}\right)=1$ if ${\hat{\alpha }}_{k,n}=\alpha_{k,n}$ and zero otherwise.

**Figure 2:**
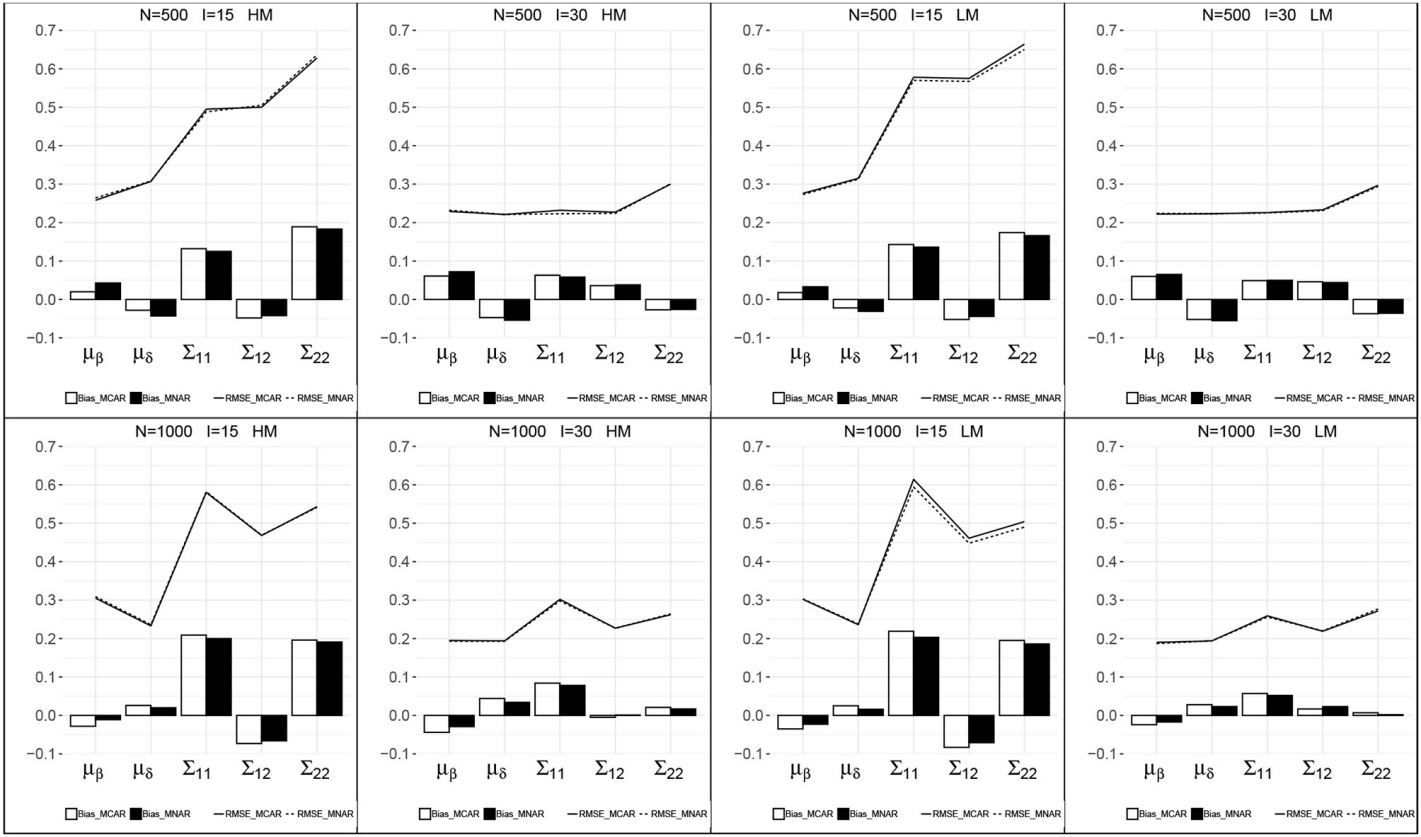
Recovery of the parameters in the item mean vector and the item covariance matrix. HM, high missingness; LM, low missingness.

Figure 2 presents the recovery of the item mean vector and the item covariance matrix for the models with MCAR and MNAR in all eight conditions. First, the patterns of the item parameter recovery were similar between MCAR and MNAR, especially the RMSEs of MCAR and MNAR were close in each condition. The item mean vector can be well recovered, as the bias were small and the RMSEs were relatively smaller than those of the item covariance matrix. The bias of the item mean vector under MNAR were smaller than those under MCAR at a higher sample size *N* = 1, 000. The RMSEs of the item mean vector decreased as test length increased. The bias and RMSEs of the item covariance matrix were relatively higher, and decreased as test length increased. The sample size had no consistent impact on the estimates of the item covariance matrix. Moreover, the missingness probabilities had little impact on the item parameter recovery.

Table 1 summarizes the item parameter recovery for models with MCAR and MNAR in high missingness conditions. The results for low missingness are similar and presented in Supplementary Figure 1. The mean absolute value (MAV) of the bias and RMSE are reported. In each condition, the MAVs of the bias and RMSE under MCAR and MNAR were close. Detailed information about the recovery of each item parameter with MCAR and MNAR was similar and not reported here.

**Table 1:** Summary of the item parameters for high missingness conditions.

		**β**				**δ**			
		**Bias**		**RMSE**		**Bias**		**RMSE**	
***I***	***N***	**MAR**	**MNAR**	**MAR**	**MNAR**	**MAR**	**MNAR**	**MAR**	**MNAR**
15	500	0.058	0.067	0.398	0.400	0.062	0.070	0.500	0.502
	1,000	0.056	0.047	0.355	0.355	0.063	0.057	0.407	0.407
30	500	0.053	0.054	0.319	0.318	0.061	0.059	0.406	0.405
	1,000	0.043	0.037	0.241	0.239	0.049	0.045	0.306	0.304

Table 2 summarizes the estimation of general ability parameter in high missingness conditions. The results for low missingness are similar and presented in Supplementary Figure 2. The MAV and Range of the bias and RMSE are reported. The correlation between the true and the estimated general abilities is also given. The MAVs of the bias under MCAR were much higher than those under MNAR, and the bias under MCAR were all negative, which can be seen from their Ranges. The MAVs of the RMSEs under MCAR were also higher than those under MNAR. The correlations under MNAR were higher than those under MAR. From the above results, we find that when data are generated with MNAR, incorrect use of missing data mechanism could lead to biased estimation of the general abilities.

**Table 2:** Summary of the general ability for high missingness conditions.

		**Bias**				**RMSE**				**Cor**	
		**MAV**		**Range**		**MAV**		**Range**			
**I**	**N**	**MCAR**	**MNAR**	**MCAR**	**MNAR**	**MCAR**	**MNAR**	**MCAR**	**MNAR**	**MCAR**	**MNAR**
15	500	0.702	0.094	(−1.130, −0.311)	(−0.342, 0.387)	0.966	0.656	(0.680, 1.286)	(0.382, 1.012)	0.691	0.782
	1,000	0.699	0.095	(−1.036, −0.291)	(−0.283, 0.366)	0.965	0.647	(0.674, 1.335)	(0.347, 1.045)	0.690	0.728
30	500	0.707	0.090	(−1.066, −0.328)	(−0.283, 0.382)	0.943	0.610	(0.610, 1.272)	(0.403, 0.873)	0.730	0.766
	1,000	0.707	0.085	(−1.078, −0.344)	(−0.404, 0.323)	0.939	0.588	(0.649, 1.230)	(0.346, 0.899	0.742	0.776

*MAV, mean absolute value; Range = (minimum, maximum)*.

**Figure 3:**
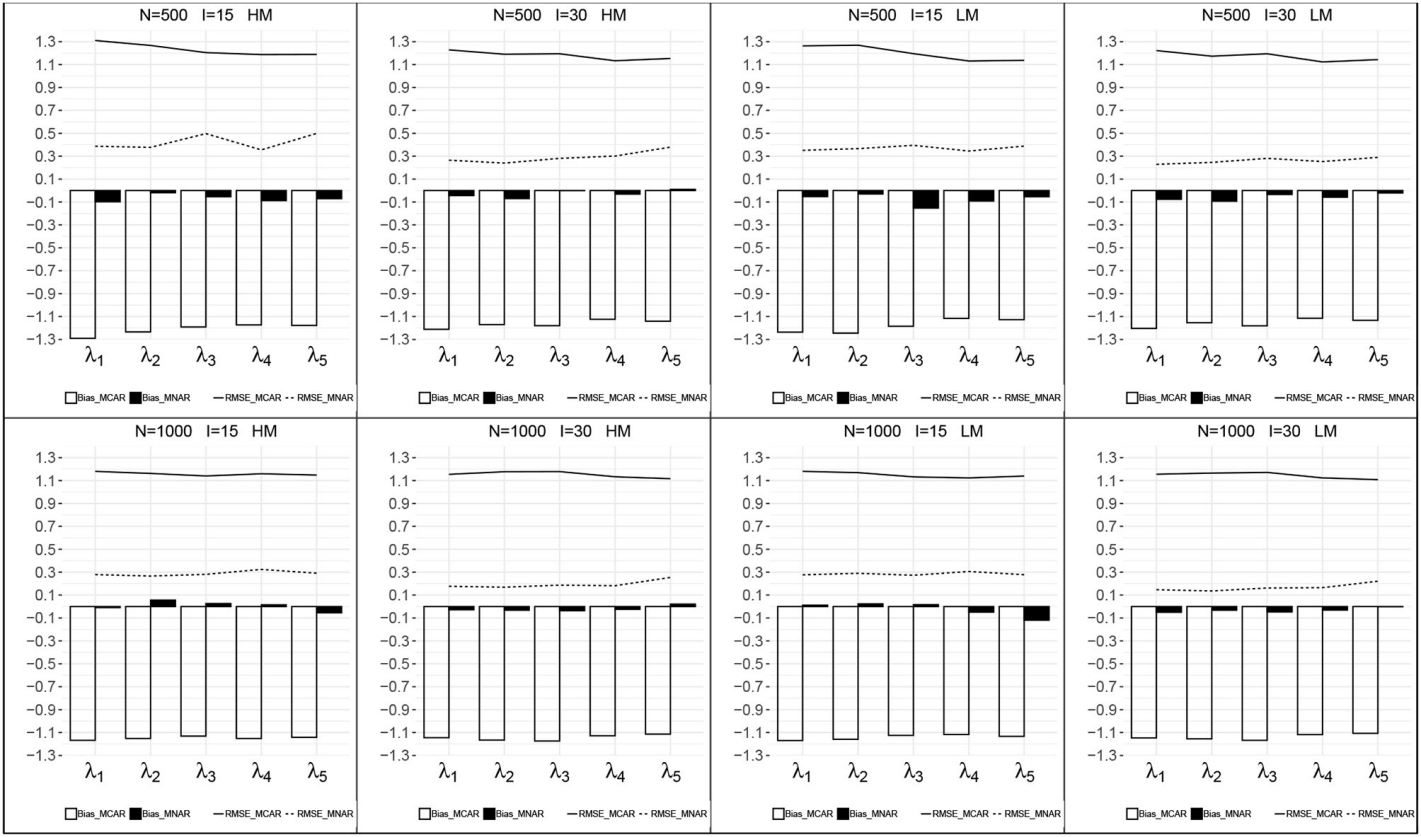
Recovery of the attribute intercept parameters. HM, high missingness; LM, low missingness.

**Figure 4:**
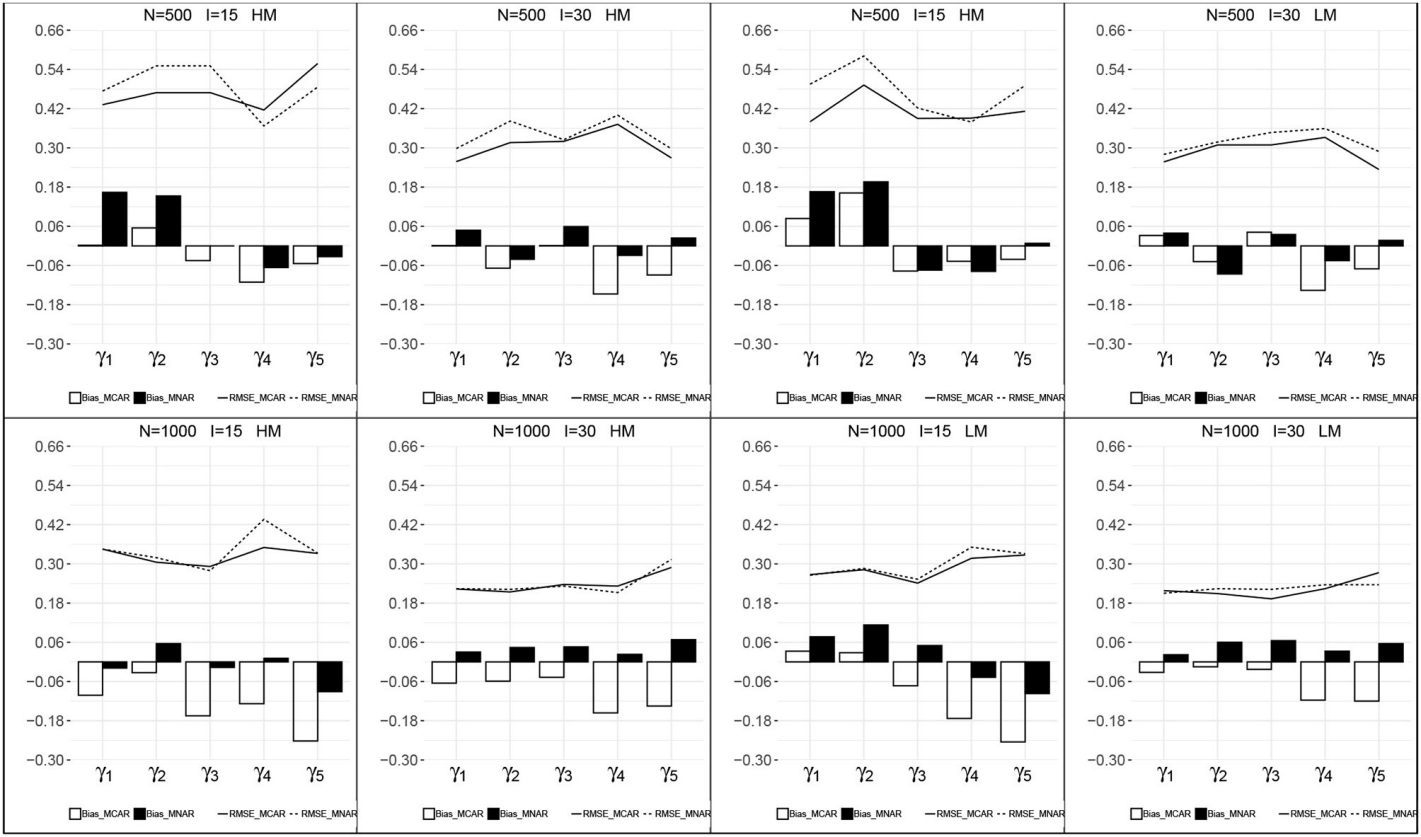
Recovery of the attribute slope parameters. HM, high missingness; LM, low missingness.

Figures 3, 4 presents the recovery of higher-order parameters between the attributes and the general ability in each condition. For the attribute slope parameters, the bias was closer to zero and the RMSEs were relatively small. For the attribute intercept parameters, their recovery under MNAR were good with small bias and RMSEs. Under MCAR, the bias and RMSEs for attribute intercept parameters were large, with absolute values >1.0, and all the bias were negative.

**Figure 5:**
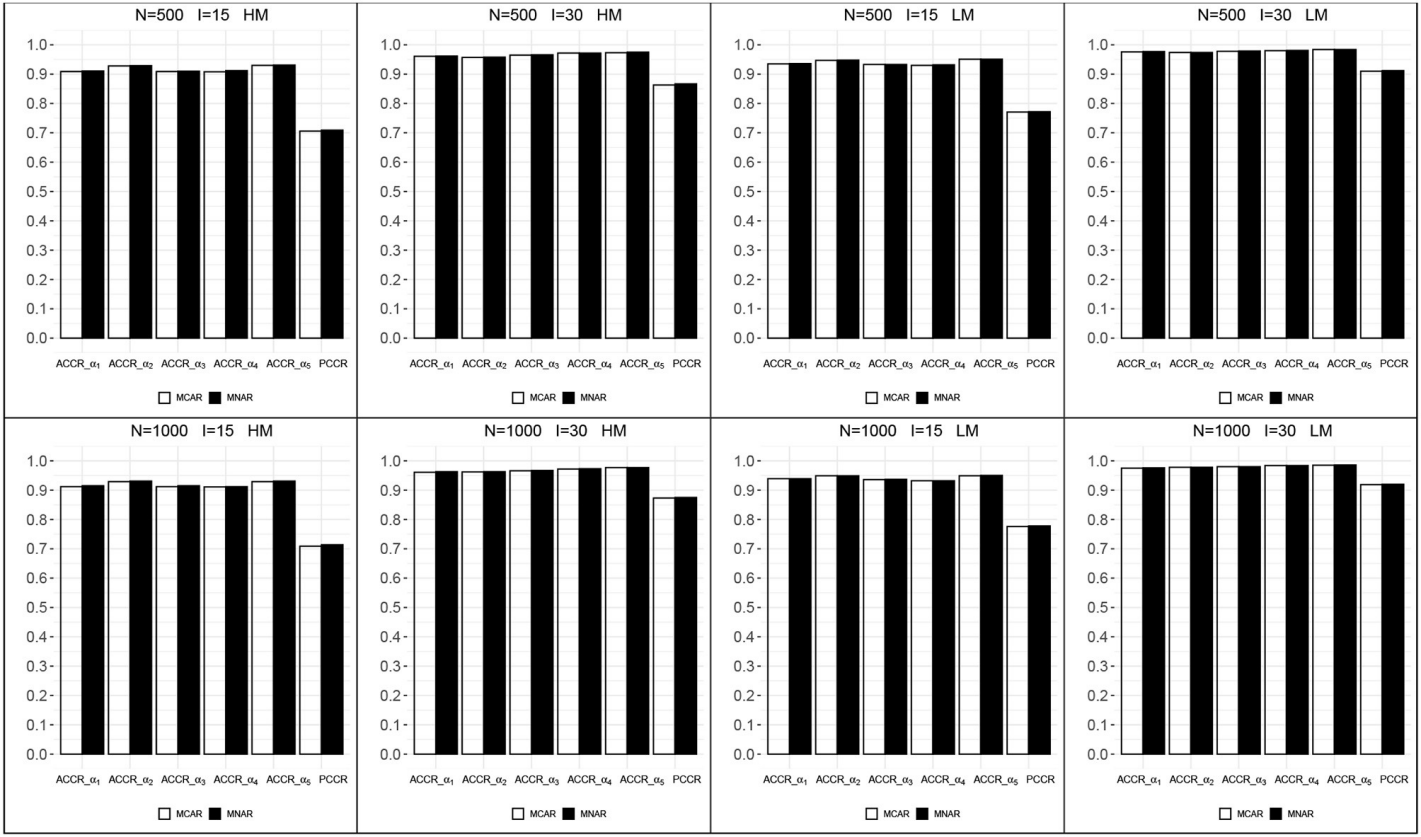
The correct classification rates for each attribute and attribute profiles. HM, high missingness; LM, low missingness.

Figure 5 shows the correct classification rates for each attribute and attribute profiles in all conditions. All ACCRs were > 0.90 and all PCCRs were > 0.70, which indicated a good recovery of the mastery status of attributes. ACCRs and PCCRs raised as test length increased and changed little as sample size increased. The correct classification rates of MCAR and MNAR were very close in each condition. These results may be caused by the fact that the impact of the missing data mechanism on the latent attributes is indirect through the general high-order ability, and we assume the same model for item response under both missing data mechanisms.

### 3.2. Simulation Study 2

The aim of Simulation 2 was to empirically examine the sensitivity of incorrect use of MNAR for model fit. In this study, the simulated data were generated with MCAR, i.e., the distribution of the general higher-order ability is independent of the latent missingness classes, and we set θ_*n*_ ~ *N*(0, 1) for identification. The other settings were the same as those used for *N* = 500, *J* = 15 and low missingness in Simulation 1, which is a weak condition studied in Simulation Study 1.

Table 3 presents the bias and RMSE for the item mean vector, the item covariance matrix and the higher-order structure parameters. The recovery of the item parameter mean vector and covariance matrix were similar under MCAR and MNAR. For the higher-order structure parameters, unlike the results in Simulation Study 1, the recovery of the parameters under MNAR were as good as those under MCAR.

**Table 3:** Recovery of the item mean vector, the item covariance matrix, the attribute slope, and intercept.

	**Index**	**μ_β_**	**μ_δ_**	**Σ_11_**	**Σ_12_**	**Σ_22_**	**λ_1_**	**λ_2_**	**λ_3_**	**λ_4_**	**λ_5_**	**γ_1_**	**γ_2_**	**γ_3_**	**γ_4_**	**γ_5_**
MAR	Bias	0.043	−0.005	0.080	0.028	0.158	0.001	−0.083	0.025	0.046	0.055	0.092	0.096	0.136	0.095	0.114
	RMSE	0.265	0.263	0.394	0.357	0.454	0.271	0.221	0.232	0.278	0.230	0.487	0.552	0.438	0.372	0.367
MNAR	Bias	0.045	−0.008	0.084	0.020	0.168	−0.052	−0.122	−0.028	0.019	0.024	0.070	0.062	0.104	0.067	0.095
	RMSE	0.263	0.261	0.399	0.368	0.469	0.346	0.280	0.299	0.342	0.317	0.494	0.624	0.462	0.384	0.386

Table 4 summarizes the recovery of the item and the general ability parameter, where the mean, standard deviation, minimum, and maximum of the bias and RMSE are reported. It also shows the correct classification rates for each attribute and attribute profiles. The results under MCAR and MNAR were similar, with mean bias close to zero and approximately equal correct classification rates for the recovery of each attribute and attribute profile. From Tables 3, 4, we found that when the data were generated under MCAR, the missing data mechanism used in the model fit had little impact on the results for diagnostic classification in our framework.

**Table 4:** Summary of the item parameter, general ability, and attributes.

	**Index**		**β**	**δ**	**θ**		**α_1_**	**α_2_**	**α_3_**	**α_4_**	**α_5_**
MAR	Bias	Mean	0.036	0.054	0.100	ACCR	0.863	0.891	0.876	0.885	0.920
		SD	0.040	0.062	0.126	PCCR	0.595				
		Min.	−0.030	−0.077	−0.388						
		Max.	0.110	0.138	0.464						
	RMSE	Mean	0.343	0.510	0.683						
		SD	0.177	0.131	0.097						
		Min.	0.175	0.401	0.431						
		Max.	0.853	0.909	1.060						
	Cor.				0.722						
MNAR	Bias	Mean	0.028	0.027	0.111	ACCR	0.864	0.891	0.875	0.885	0.921
		SD	0.045	0.067	0.129	PCCR	0.594				
		Min.	−0.041	−0.091	−0.425						
		Max.	0.112	0.141	0.476						
	RMSE	Mean	0.340	0.509	0.716						
		SD	0.171	0.124	0.097						
		Min.	0.171	0.399	0.462						
		Max.	0.823	0.871	1.049						
	Cor.				0.720						

*SD, standard deviation; Min., minimum; Max., maximum; Cor., correlation between true and estimated values*.

## 4. Real Data Analysis

To illustrate the application of the proposed method, the PISA 2015 computer-based mathematics data were used. The data include item scores and response times for each item, and we only select dichotomous item scores for illustration. Four attributes belonging to the mathematical content knowledge were evaluated, i.e., change and relationship (α_1_), quantity (α_2_), space and shape (α_3_) and uncertainty and data (α_4_). *I* = 9 items with dichotomous responses were selected, with items IDs CM033Q01, CM474Q01, CM155Q01, CM155Q04, CM411Q01, CM411Q02, CM803Q01, CM442Q02, and CM034Q01. The Q matrix was shown in Table 5. Item responses with code 0 (no credit), code 1 (full credit), and code 9 (noresponse) were considered here. The responses “nonresponse” (code 9) were treated as missing data and might be due to a MNAR mechanism. *N* = 758 test-takers from Albania with responses 0, 1, and 9 to each of the nine items were used for analysis. The missing rate (i.e., the proportion of “nonresponse”) for each item ranged from 0 to 14.78%. The models with missing data mechanisms MCAR and MNAR were both fitted to the data. The number of latent missingess classes *C* = 2 was determined by latent class analysis. Then, the analysis was specified in the same way as the simulation study. The DIC was applied to compare model fit for models under different missing data mechanisms.

**Table 5:** The *Q* matrix in the real data.

**Attribute**	**CM033Q01**	**CM474Q01**	**CM155Q01**	**CM155Q04**	**CM411Q01**	**CM411Q02**	**CM803Q01**	**CM442Q02**	**CM034Q01**
α_1_	0	0	1	1	0	0	0	0	0
α_2_	1	0	0	0	0	0	0	0	1
α_3_	0	1	0	0	1	0	0	1	0
α_4_	0	0	0	0	0	1	1	0	0

The DIC values under MCAR and MNAR were 11454.2 and 10406.3, respectively, which indicated that MNAR was preferred with a lower DIC value. We were only interested in the results concerning diagnostic classification. Table 6 reports the estimated parameters and its corresponding standard deviations for the item mean vector, the item covariance matrix, and the attribute intercept and slope parameters. The results for the item mean vector and covariance matrix were similar under different missing data mechanisms, and the estimated Σ_12_ was −1.069, which indicated that items with a higher intercept corresponded to a lower interaction. μ_β_ was estimated to be −2.033, which means that the mean guessing probability was approximate 0.12. All estimated attribute intercept and slope parameters were positive. The estimations for the item covariance matrix and the attribute intercept and slope parameters were poor, which were consistent with previous studies (de la Torre and Douglas, 2004; Zhan et al., 2018a). Table 7 reports the estimated item parameters. All δ_*i*_ were positive, which means that all items satisfy *g*_*i*_ < 1 − *s*_*i*_. Only β_*i*_ for CM033Q01 was positive, which means that the guessing probability of this item is higher than 0.5.

**Table 6:** Estimates and standard errors of the parameters for the real data.

	**DIC**	**Index**	**μ_β_**	**μ_δ_**	**Σ_11_**	**Σ_12_**	**Σ_22_**	**λ_1_**	**λ_2_**	**λ_3_**	**λ_4_**	**γ_1_**	**γ_2_**	**γ_3_**	**γ_4_**
MCAR	11454.2	Est.	−2.033	2.548	3.383	−1.069	1.451	0.677	2.051	1.421	1.888	3.843	3.795	4.208	3.259
		SD	0.587	0.426	2.341	1.411	1.195	0.688	0.845	0.710	0.866	1.082	0.891	0.990	0.912
MNAR	10406.3	Est.	−2.073	2.419	3.313	−1.105	1.269	2.828	2.653	3.055	3.852	2.261	1.787	2.099	1.675
		SD	0.572	0.391	2.280	1.305	1.087	0.966	0.901	0.994	1.317	0.862	0.778	0.683	0.702

*Est., Estimated values; SD, standard deviation of the posterior distribution*.

**Table 7:** Estimates and standard errors of the parameters for the real data.

	**Par**.	**033Q01**	**474Q01**	**155Q01**	**155Q04**	**411Q01**	**411Q02**	**803Q01**	**442Q02**	**034Q01**
MCAR	β_*i*_	0.155	−0.501	−0.665	−1.445	−1.737	−1.355	−4.227	−4.587	−2.945
		(0.139)	(0.156)	(0.289)	(0.185)	(0.256)	(0.166)	(0.811)	(0.846)	(0.412)
	δ_*i*_	2.447	1.589	3.342	1.588	2.508	0.788	3.458	3.219	2.775
		(0.588)	(0.256)	(0.678)	(0.262)	(0.333)	(0.304)	(0.824)	(0.869)	(0.456)
MNAR	β_*i*_	0.019	−0.579	−0.676	−1.452	−1.761	−1.321	−4.073	−4.543	−3.347
		(0.151)	(0.145)	(0.245)	(0.164)	(0.244)	(0.139)	(0.795)	(0.740)	(0.575)
	δ_*i*_	2.081	1.655	3.068	1.588	2.316	0.733	3.308	3.042	2.881
		(0.432)	(0.233)	(0.500)	(0.228)	(0.295)	(0.277)	(0.809)	(0.747)	(0.570)

*Par., estimated parameter; the first two letters for item IDs (i.e., “CM”) are omitted to save space*.

**Figure 6:**
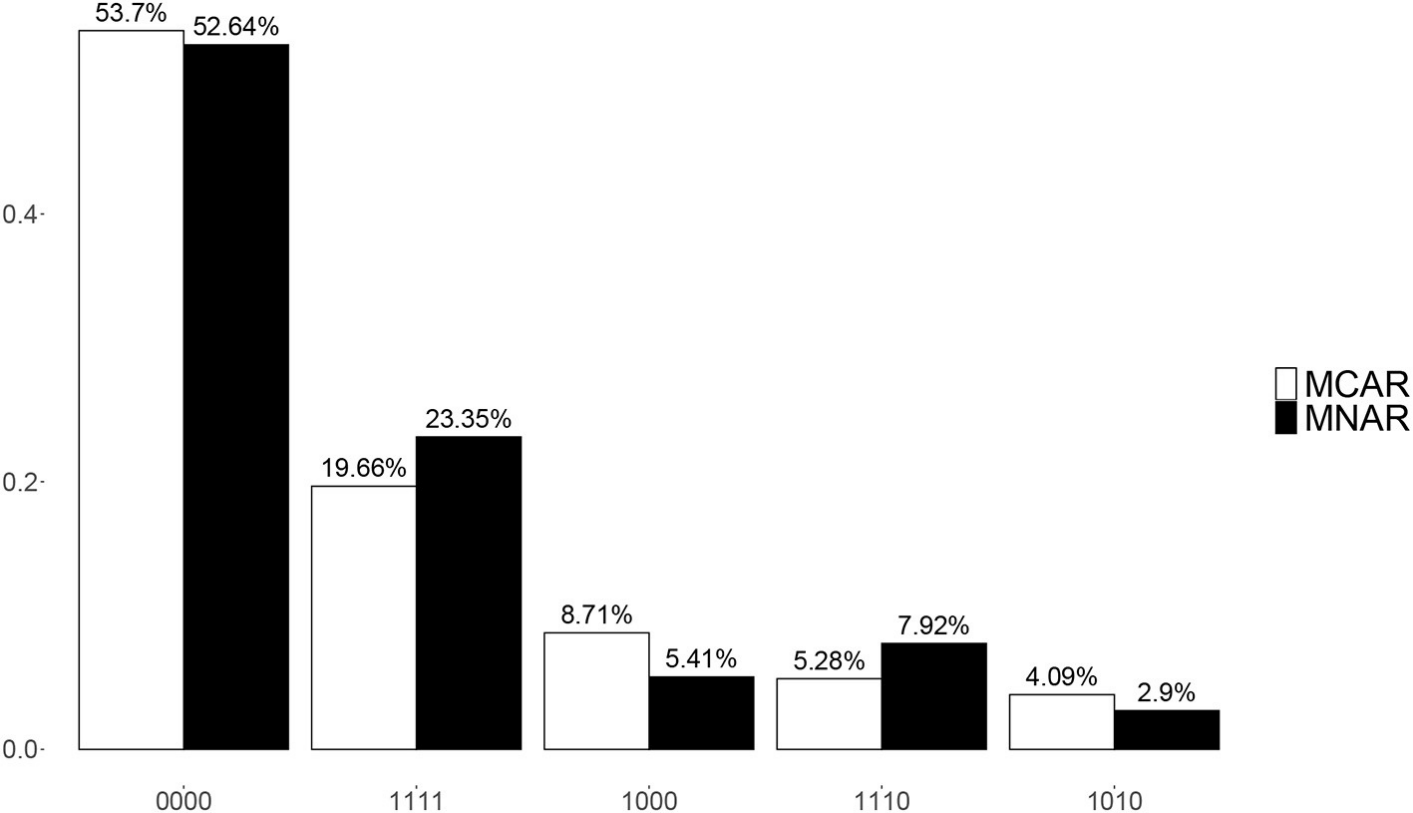
Posterior mixing proportions for top 5 most frequent attribute patterns under MCAR and MNAR in the real data.

Though there were 16 possible attribute patterns for four attributes, 15 attribute patterns except (0101) were found in the estimated attributes under both MCAR and MNAR. Figure 6 presents the top five most frequent attribute patterns in the real data under MCAR and MNAR. The most prevalent attribute pattern was (0000) and the second most prevalent pattern was (1111) under both MCAR and MNAR, where the corresponding proportions were slightly different. The third and the fourth most prevalent patterns under MCAR and MNAR were reverse. The above results indicate that the missing data mechanisms have some influences on the estimated attribute patterns.

## 5. Conclusions

When multiple items are used to classify subjects' mastery status of latent attributes, it is almost inevitable that item-level missing data will occur. It is possible that the missing data mechanism is related to the item responses, and without very strong assumptions, item-level non-response can be thought to depend on some latent variables. Motivated by this idea, we have proposed a joint modeling method incorporating item responses and missing data mechanism for cognitive diagnosis. A latent categorical variable is employed to describe the latent missingness propensity, which can avoid distributional assumptions and result in a more flexible model. Then, the latent missingness classes are linked to each item missingness indicator by the logistic function. Applying the hierarchical modeling framework, the general higher-order ability's distribution is affected by the latent missingness class in the case of MNAR and is independent of the latent missingness class in the case of MCAR.

A Bayesian MCMC method is used to estimate the model parameters under the missing data mechanisms MCAR and MNAR. The simulation study demonstrates that when the data are generated under MNAR, the estimated general ability and the attribute intercept parameters have higher bias if an incorrect missing data mechanism is used for model fit; when the data are generated under MCAR, the results between different missing data mechanisms do not have much difference. Similar results about the impact of the missing data mechanisms have been found by de Chiusole et al. (2015) in the framework of KST. The PISA 2015 computer-based mathematics data are used to explore the magnitude and direction of item and person parameters, and the results support the MNAR in the real data analysis.

Our proposed method can be further investigated in several aspects. First, the proposed model has good performance with DINA model used for illustration, and the joint modeling method can be extended to other types of CDMs for further studies. Second, this study assumes that each latent attribute is a binary variable. When polytomous attributes are involved in CDMs (Zhan et al., 2019b), modified higher-order CDMs could be utilized in the framework of our model. Third, multiple sources of data about a subject behavior, for example, response time and other process data, can be combined to build up a more general model, which could provide a more comprehensive reflection of individual behavior. Finally, the number of latent missingness classes can be varied. For selecting it, Akaike information criterion (AIC), Bayesian information criterion (BIC) or DIC, can be utilized to compare different models under various choices.

## Data Availability Statement

Publicly available datasets were analyzed in this study. This data can be found at: http://www.oecd.org/pisa.

## Author Contributions

NS provided original idea of the paper. XW provided the technical support. Both authors contributed to the article and approved the submitted version.

## Conflict of Interest

The authors declare that the research was conducted in the absence of any commercial or financial relationships that could be construed as a potential conflict of interest.
