# Supplementary material for: Cognitive Diagnosis Modeling Incorporating Item-Level Missing Data Mechanism
Source: Front Psychol. 2020 Nov 30;11:564707. doi: 10.3389/fpsyg.2020.564707 (PMC7733994; doi:10.3389/fpsyg.2020.564707)
Supplement: Supplementary file 1 [file Data_Sheet_1.PDF]

Supplementary Material

1 SAMPLE JAGS CODE FOR MODEL UNDER MNAR

```
model{
  for(n in 1:N){
    for(i in 1:I){
      for(k in 1:K){w[n, i, k] <- pow(attribute[n, k], Q[i, k])}
    logit(prob[n, i]) <- beta1[i] + delta1[i] * prod(w[n, i, ])
    Score[n, i] ~ dbern(prob[n, i])
    logit(prR[n,i]) <- b0[i] + bt[R1[n],i]
    R[n,i] ~ dbern(prR[n,i])
    }
  }
  for(n in 1:N){
    for(k in 1:K){
      logit(att_prob[n, k]) <- gamma[k] * theta[n] - lambda[k]
      attribute[n, k] ~ dbern(att_prob[n, k])
    }
    theta[n] ~ dnorm(mu_theta[R1[n]], den_theta[R1[n]])
  }
  probA[1:C] ~ ddirch(alpha[1:C])
  for(n in 1:N){R1[n] ~ dcat(probA[1:C])}
  for(k in 1:K){
    lambda[k] ~ dnorm(0, 0.25)
    gamma[k] ~ dnorm(0, 0.25)%_% T(0, )
  }
  for(i in 1:I){
    item_parameter[i, 1:2] ~ dmnorm(item_mu[1:2], item_den[1:2, 1:2])
    beta1[i] <- item_parameter[i, 1]
    delta1[i] <- item_parameter[i, 2]
  }
  alpha[1] <- 1
  alpha[2] <- 1
  mu_theta[1] <- 0
  mu_theta[2] ~ dnorm(0, 0.25)
  den_theta[1] <- 1
  den_theta[2] ~ dgamma(1,1)
  item_mu[1] ~ dnorm(-2.197, 0.5)
  item_mu[2] ~ dnorm(4.394, 0.5)%_% T(0, )
  R0[1, 1] <- 1
  R0[2, 2] <- 1
  R0[1, 2] <- 0
  R0[2, 1] <- 0
```

```
  item_den[1:2, 1:2] ~ dwish(R0[1:2, 1:2], 2)
  Sigma_item[1:2, 1:2] <- inverse(item_den[1:2, 1:2])
  for(i in 1:I){
    b0[i] ~ dnorm(0, 0.25)
    bt[1,i]<-0
     for(c in 2:C){
       bt[c,i] ~ dnorm(0, 0.25)%_% T(0, )
     }
  }
}
```

2 SUPPLEMENTARY TABLES

Table S1. Summary of the item parameters for low missingness conditions in Simulation Study 1

		β				δ			
		Bias		RMSE		Bias		RMSE	
I	N	MAR	MNAR	MAR	MNAR	MAR	MNAR	MAR	MNAR
15	500	0.044	0.049	0.377	0.373	0.062	0.066	0.467	0.462
	1000	0.056	0.044	0.307	0.301	0.070	0.061	0.362	0.357
30	500	0.036	0.035	0.277	0.277	0.048	0.045	0.367	0.366
	1000	0.030	0.031	0.198	0.199	0.041	0.041	0.265	0.266

Table S2. Summary of the general ability for low missingness conditions in Simulation Study 1

		Bias				RMSE				Cor	
		MAV		Range		MAV		Range			
I	N	MCAR	MNAR	MCAR	MNAR	MCAR	MNAR	MCAR	MNAR	MCAR	MNAR
15	500	0.702	0.093	(-1.133,-0.323)	(-0.383,0.381)	0.950	0.668	(0.673,1.303)	(0.437,0.965)	0.717	0.737
	1000	0.699	0.087	(-1.040,-0.272)	(-0.309,0.409)	0.947	0.640	(0.668,1.295)	(0.340,0.994)	0.718	0.740
30	500	0.707	0.089	(-1.065,-0.346)	(-0.309,0.382)	0.934	0.610	(0.623,1.258)	(0.411,0.903)	0.744	0.766
	1000	0.707	0.087	(-1.037,-0.334)	(-0.372,0.366)	0.931	0.590	(0.637,1.253)	(0.324,0.857)	0.754	0.777

Note. MAV = mean absolute value; Range = (minimum, maximum).

In the following, some results for a simulation study with $C = 3$ are presented. The test length is $I = 15$, and the sample size $N = 1000$ is used since more parameters are involved in this simulation. Most of the parameter settings were the same to those used in Simulation Study 1. The different parameter settings for $C = 3$ were given below. $\boldsymbol{\pi} = (0.1, 0.3, 0.6)$, representing unequal probabilities for each latent class; $\tau_{0i} = (0.0, 0.5, 0.0, 0.5, 0.0, 0.5, 0.0, 0.5, 0.0, 0.5, 0.0, 0.5, 0.0, 0.5, 0.0)$, $\tau_{2i} = 1.2$ and $\tau_{3i} = 2.4$ for all items; $\mu_2 = 1.0$, $\mu_3 = 2.0$, $\sigma_2^2 = 0.5$ and $\sigma_3^2 = 2.0$. Other assignments of the model parameters also make sense, and will lead to similar results. Table S3 presents the recovery of the item mean vector, the item covariance matrix, the attribute slope and intercept for the models with MCAR and MNAR. Table S4 summarizes the item parameter, general ability and attributes. The results are similar to those in Simulation Study 1 with $C = 2$.

Table S3. Recovery of the item mean vector, the item covariance matrix, the attribute slope and intercept for $C = 3$

	Index	μ_β	μ_δ	Σ_{11}	Σ_{12}	Σ_{22}	λ_1	λ_2	λ_3	λ_4	λ_5	γ_1	γ_2	γ_3	γ_4	γ_5
MAR	Bias	0.044	-0.065	0.280	-0.110	0.131	-2.167	-2.210	-2.161	-2.164	-2.219	0.368	0.404	0.335	0.299	0.518
	RMSE	0.251	0.258	0.614	0.470	0.495	2.193	2.229	2.169	2.170	2.225	0.523	0.525	0.461	0.438	0.633
MNAR	Bias	0.062	-0.074	0.266	-0.096	0.118	-0.334	-0.380	-0.381	-0.443	-0.290	-0.006	-0.051	-0.140	-0.180	0.003
	RMSE	0.251	0.255	0.582	0.441	0.471	0.552	0.486	0.550	0.545	0.473	0.413	0.366	0.296	0.340	0.394

Table S4. Summary of the item parameter, general ability and attributes for $C = 3$

	Index		β	δ	θ		α_1	α_2	α_3	α_4	α_5
MAR	Bias	Mean	-0.008	0.020	-1.508	ACCR	0.953	0.962	0.947	0.944	0.956
		SD	0.057	0.068	0.180	PCCR	0.824				
		Min.	-0.096	-0.110	-2.167						
		Max.	0.124	0.147	-0.946						
	RMSE	Mean	0.353	0.380	1.796						
		SD	0.158	0.146	0.197						
		Min.	0.174	0.228	1.131						
		Max.	0.691	0.714	2.387						
	Cor.				0.712						
MNAR	Bias	Mean	0.013	0.009	-0.075	ACCR	0.954	0.962	0.948	0.946	0.958
		SD	0.055	0.064	0.173	PCCR	0.826				
		Min.	-0.066	-0.117	-0.639						
		Max.	0.144	0.114	0.547						
	RMSE	Mean	0.351	0.377	0.998						
		SD	0.152	0.139	0.151						
		Min.	0.182	0.234	0.599						
		Max.	0.684	0.702	1.796						
	Cor.				0.740						

Note. SD = standard deviation; Min. = minimum; Max. = maximum; Cor. = correlation between true and estimated values.
